# Selection of human induced pluripotent stem cells lines optimization of cardiomyocytes differentiation in an integrated suspension microcarrier bioreactor

**DOI:** 10.1186/s13287-020-01618-6

**Published:** 2020-03-13

**Authors:** Filip Laco, Alan Tin-Lun Lam, Tsung-Liang Woo, Gerine Tong, Valerie Ho, Poh-Loong Soong, Elina Grishina, Kun-Han Lin, Shaul Reuveny, Steve Kah-Weng Oh

**Affiliations:** 1 Bioprocessing Technology Institute, 20 Biopolis Way, Centros #06-01, Singapore, 138668 Singapore; 2 Ternion Biosciences, National Heart Centre of Singapore, Singapore, Singapore

**Keywords:** Bioprocessing, Cardiomyocytes, Human induced pluripotent stem cells, Microcarriers, Stirred tank bioreactor, OptioQUANT™ platform

## Abstract

**Background:**

The production of large quantities of cardiomyocyte is essential for the needs of cellular therapies. This study describes the selection of a human-induced pluripotent cell (hiPSC) line suitable for production of cardiomyocytes in a fully integrated bioprocess of stem cell expansion and differentiation in microcarrier stirred tank reactor.

**Methods:**

Five hiPSC lines were evaluated first for their cardiac differentiation efficiency in monolayer cultures followed by their expansion and differentiation compatibility in microcarrier (MC) cultures under continuous stirring conditions.

**Results:**

Three cell lines were highly cardiogenic but only one (FR202) of them was successfully expanded on continuous stirring MC cultures. FR202 was thus selected for cardiac differentiation in a 22-day integrated bioprocess under continuous stirring in a stirred tank bioreactor. In summary, we integrated a MC-based hiPSC expansion (phase 1), CHIR99021-induced cardiomyocyte differentiation step (phase 2), purification using the lactate-based treatment (phase 3) and cell recovery step (phase 4) into one process in one bioreactor, under restricted oxygen control (< 30% DO) and continuous stirring with periodic batch-type media exchanges. High density of undifferentiated hiPSC (2 ± 0.4 × 10^6^ cells/mL) was achieved in the expansion phase. By controlling the stirring speed and DO levels in the bioreactor cultures, 7.36 ± 1.2 × 10^6^ cells/mL cardiomyocytes with > 80% Troponin T were generated in the CHIR99021-induced differentiation phase. By adding lactate in glucose-free purification media, the purity of cardiomyocytes was enhanced (> 90% Troponin T), with minor cell loss as indicated by the increase in sub-G1 phase and the decrease of aggregate sizes. Lastly, we found that the recovery period is important for generating purer and functional cardiomyocytes (> 96% Troponin T). Three independent runs in a 300-ml working volume confirmed the robustness of this process.

**Conclusion:**

A streamlined and controllable platform for large quantity manufacturing of pure functional atrial, ventricular and nodal cardiomyocytes on MCs in conventional-type stirred tank bioreactors was established, which can be further scaled up and translated to a good manufacturing practice-compliant production process, to fulfill the quantity requirements of the cellular therapeutic industry.

**Supplementary information:**

The online version of this article (10.1186/s13287-020-01618-6) contains supplementary material, which is available to authorized users.

## Introduction

One of the leading causes of death worldwide has been cardiac-related illnesses, such as myocardial infarction, also known as heart attack [22]. Inability of mature cardiomyocytes (CMs) to regenerate is the main cause leading to heart failure [15, 22]. Because of the limited regenerative potential of the cardiac cells, heart transplantation for reconstituting the function of damaged heart is the only effective solution currently available [22]. This is, however, severely hindered mainly due to the shortage of donor organs [15, 22]. To that end, stem cell-derived CMs may serve as a renewable cellular source for repairing the infracted myocardium.

Human pluripotent stem cells (hPSCs) and human induced-pluripotent stem cells (hiPSCs) are characterized by their extensive proliferative capacity and their ability to differentiate towards functional CMs [2, 10, 53]. However, it is suggested that doses of up to 1 billion functional CMs are required for a single cell therapy for an infarcted myocardium [15, 16, 24]. It has been a challenge to produce large quantities of high-purity CMs due to the adherent characteristic of stem cells and their differentiation efficiency [2, 10, 22, 37]. A prerequisite for realizing the therapeutic potential of stem cells is the translation of inefficient manually operated monolayer base cell expansion and cardiac differentiation methods into an efficient, scalable, streamlined, closed system and controllable manufacturing process.

The expansion of hPSCs/hiPSCs in a spinner flask as aggregates with and without microcarriers (MCs) has been well documented [1, 6, 35] and scaled up into closed bioreactor systems [30, 54] which paved the way to commercialization and industrial manufacturing [50]. The differentiation of hPSCs/hiPSCs aggregates with and without MCs or with hydrogel [29] to CMs was also well demonstrated [37]. The scale up of the cardiac differentiation process into a fully closed system has also been well documented [37], e.g. in wave bag reactors [12] and with stirring tank reactors [27] as suspended embryoid bodies (EBs). However, the processes of making EBs are labour intensive and problematic since it requires extensive cell handling such as dissociating or cutting [31, 37]. Besides, although EBs with controlled size can be form by many methods [45, 46, 62], they might be limited to research scale [37]. In the past few years, our group has developed protocols for scale-up and production of de novo hPSC-/hiPSC-derived CMs based on the use of MC cultures in platforms such as rockers and spinners [7, 8, 34, 35, 38, 57–59]. However, in order to generate high-quantity and high-quality CMs, applying intermittent agitation during the first 3 days of cardiac differentiation phase is important [57, 58], which makes it problematic in large-scale reactors. In addition, the continuous monitoring and control of the environment within a spinner culture are still challenging. This problem becomes increasing important when cells reach high densities and pH can be highly acidic and oxygen availability to the cells can be hampered. In this study, we adapted our MC-based cardiac differentiation system from spinners [33, 58] to establish a robust, scalable and controllable process for manufacturing CMs in a single enclosed industrial-type stirred tank reactor. Stirred tank bioreactors are commonly used for the production of cellular products, such as antibodies, enzymes, vaccines and virus in the biotechnology industry. It offers several advantages like ease of design, scale-up and operation in different batch modes including the ability to integrate online monitoring probes and control of culture variables, such as pH, dissolved oxygen (DO), nutrients, temperature and metabolites [26, 48].

Other than controlling the bioprocess parameters to enhance the efficiency of cardiac differentiation, selection of the best cell line with high cardiac differentiation potential is also critical for CM production. Differences in cell lines have been shown to affect cardiac differentiation efficiency and cellular growth [32]. S cell-cycle phase was correlated with the cardiac differentiation efficiency. High cardiac differentiation efficiency was observed in high S/G2/M phases with low dose of CHIR99021 [32].

In this study, we attempt to integrate the phases of undifferentiated hiPSC expansion, CHIR99021-induced cardiac differentiation and lactate purification into a scalable MC suspension culture system using a stirred tank bioreactor under continuous agitated condition, providing a streamlined and controlled process for scale-up of pure CM production. Lower CM yields previously seen in a spinner flask under continuous agitation [57, 58] were corrected with the process of cell line selection and by controlling the culture parameters in the bioreactor. A process for CM production that is amenable to good manufacturing practice (GMP) and scale-up was developed. CMs were tested for their physiological functions using a novel high-throughput OptioQUANT™ platform (Ternion Bioscience, Singapore).

## Materials and methods

### Culture of hiPSCs in monolayer cultures

The human-induced pluripotent stem cell lines iPS (IMR90)-1 (IMR90), iPS-DF6-9-9T (DF6), IISH1i-BM1 (BM1) and IISH3i-CB6 (CB6) (WiCell Research Institute, USA) and FR202 (lung fibroblast-derived iPSC in our lab [32]) were cultured on Geltrex® (Thermo Fisher Scientific, USA) coated tissue culture plates with mTeSR™1 (Stemcell Technologies, Canada). Media was refreshed daily, and cultures were passaged with ReLeSR™ (Stemcell Technologies, Canada) according to the manufacturer’s description every 3–4 days at about 50–70% cell plate confluences. Passage was done at 1:10–15 split ratio. Cell cultures were incubated at 37 °C in a humidified atmosphere with 5% CO_2_.

### Cardiac differentiation of hiPSCs in monolayer culture

Protocol was adapted from Lian et al. [40] with some modifications as we previously reported [32]. Briefly, hiPSCs were grown until 70–90% confluence in six-well plates. Culture media was replaced with the differentiation medium RPMI 1640 + B-27™ Supplement (without insulin) (Thermo Fisher Scientific, USA) + 0.6 mM L-ascorbic acid 2-phosphate (Sigma-Aldrich, USA), designated as RPMI/B27^-IN^, and GSK3β inhibitor CHIR99021 (Selleckchem, USA) 4-12 μM was added [32]. The differentiation medium was changed after 24 h in order to remove/reduce CHIR concentration to less than 1.5 μM. Wnt inhibitor IWR-1 (Selleckchem, USA) 2.5 μM was added on day 2 [32] and was removed during the medium change on day 5. Cultures were maintained in the differentiation medium with the medium changed every 24 h. Cells were incubated at 37 °C in a humidified atmosphere with 5% CO_2_. All small molecules were solubilized in DMSO (Sigma-Aldrich, USA). Cardiac markers: NK2 Homeobox 5 (NKX2–5), Troponin T and Myosin light chain 2a (MLC2a); endodermal marker hepatocyte nuclear factor 4 alpha (HNF4a; and mesodermal marker (CD44) were measured by flow cytometry on day 14 differentiation.

### Preparation of microcarriers

Method for microcarrier coating have been reported earlier by our group [57, 58]. Briefly, 20 mg/mL of Cytodex 1 (GE Healthcare, USA) was coated with (1:30 v/v) Geltrex® in DMEM/F12 medium (Thermo Fisher Scientific) overnight at 4 °C. Prior to use, pre-warmed (37 °C for 45 min) Cytodex 1 was centrifuged at 2000 rpm for 5 min. The supernatant was replaced with mTeSR™1 supplemented with 10 μM ρ-activated kinase inhibitor Y-27632 (Selleckchem, USA).

### Expansion and cardiac differentiation of hiPSCs in microcarrier spinner cultures

Microcarrier spinner flask cultures were operated in a procedure similar to our previously reported study [6, 58]. Briefly, cells obtained from monolayer culture plates were seeded at cell concentration of 2 × 10^5^ cells/mL into 125-mL spinner flasks (Corning, USA) containing 25 mL medium and 50 mg pre-coated Cytodex 1 microcarriers. Spinner cultures were maintained static for 6 h. Thereafter, another 25 mL of mTeSR™1 was added, and the culture was then stirred at 25 rpm for 6 days. Eighty percent of the spent medium was replaced daily with fresh mTeSR™1 medium. Cell growth was measured daily by nuclei count using Nucleocounter NC-3000 (Chemometec, USA). The size of the cell/MC aggregates and the expression of the pluripotent markers (Tra-1-60 and Oct4a) were measured on day 6.

MC-based differentiation in the spinner flasks was performed according to protocols previously described [33, 57, 58]. Similar to the monolayer differentiation protocol aforementioned, the differentiation was initiated by replacing the spent mTeSR™1 medium with differentiation medium (RPMI+B27^-IN^) containing CHIR99021. The differentiation medium was then changed after 24 h followed by addition of IWR-1. The IWR-1 was removed during the medium change on day 5. Cultures were maintained in the differentiation medium with the medium changed every 24 h. Cells were incubated at 37 °C in a humidified atmosphere with 5% CO_2_. Cultures were harvested for cell count and the expression of Troponin T by FACS on day 12 of differentiation.

### hiPSC expansion, cardiac differentiation and purification in a 300-mL controlled bioreactor culture (Fig. 1)

The 0.5 L controlled bioreactor (Biostat B-DCU, Sartorius, Germany) containing 6 g of pre-coated Cytodex 1 microcarriers (20 mg/mL) in 300 mL of mTeSR™1 medium supplemented with 10 μM Y-27632 were seeded with FR202 cells at concentration of 1.2 ± 10^5^ cells/mL. The culture was controlled at 37 °C and agitated from 33 rpm, ensuring homogeneous suspension of microcarriers, and agitation rate was increased to 40 rpm when larger aggregates were generated. DO was maintained at 30% through overhead aeration with mixture of O_2_, N_2_ and air. The culture pH was maintained at 7.2 via the addition of CO_2_ to the gas stream. Fifty percent medium changes (150 mL) were conducted every day to prevent glucose depletion during the exponential growth phase. Cells were expanded for 5 days achieving ~ 2 × 10^6^ cells/mL and cell/MC aggregates sizes ~ 1 mm^2^ which were subjected to cardiac differentiation.

**Fig. 1:**
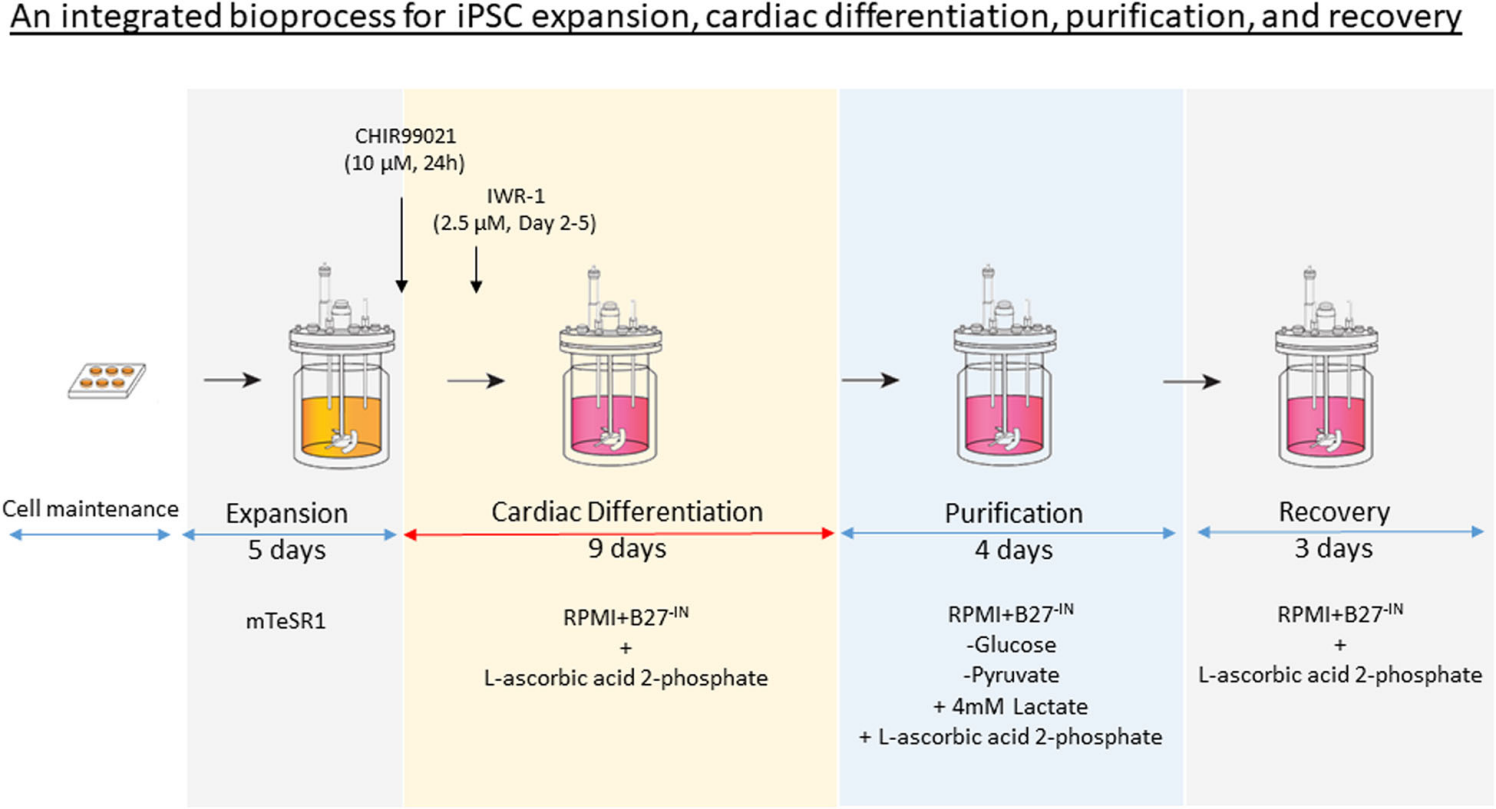
Schematic diagram demonstrating an integrated scalable one-unit bioprocess for iPSC expansion, cardiac differentiation, purification and recovery in a continuous stirring bioreactor

For cardiac differentiation, cell/MC aggregates were allowed to sediment and washed twice with 150 mL of PBS and once with 150 mL of RPMI+B27^-IN^. Thereafter, cell/MC aggregates were maintained in 300 mL of RPMI+B27^-IN^ medium supplemented with the optimized CHIR99021 concentration (10 μM for the FR202 cell line) and for 24 h at 40 rpm. Thereafter, aggregates were washed with 150 ml of RPMI+B27^-IN^ medium to remove the CHIR99021. On day 3 of differentiation, cells were treated with 2.5 μM IWR-1 in RPMI+B27^-IN^ medium. IWR-1 was removed on day 5 via a medium change with fresh RPMI+B27^-IN^. Cultures were maintained in this differentiation medium RPMI+B27^-IN^ at 40 rpm for another 3 days with 50% medium changes every 24 h.

For cardiomyocyte purification, cell/MC aggregates from day 8 of differentiation cultures (day 12 of the whole bioprocess) were washed twice with RPMI-1640 without d-glucose and pyruvate (Thermo Fisher Scientific, USA) and then re-suspended in purification medium (RPMI-1640 medium without d-glucose and pyruvate, and supplemented with 4 mM of sodium l-lactate (Sigma-Aldrich, USA) and 0.6 mM of l-ascorbic acid 2-phosphate) [58]. Cultures were maintained for 5 days at 40 rpm with 50% media changed every 24 h. Subsequently, the media was changed back to RPMI+B27^-IN^ for a 4 days’ recovery phase at 40 rpm with 50% medium changed every 24 h.

All glass bioreactors and bottles were pre-siliconized with Sigmacote® solution (Sigma-Aldrich, USA) according to the manufacturer’s protocol, to prevent any microcarriers from sticking onto the inner surface of the glass containers.

### Cell count

Cell number and cell viability was determined as previously described [33, 57, 58] by the nuclei count method using Nucleocounter NC-3000 according to the manufacturer’s instructions. Aggregate sizes were evaluated by measuring the two-dimensional area of the microscopic images of the aggregates (*n* > 50) using a Nikon *Ti*–E phase contrast microscope coupled with Nikon imaging software, NIS elements (Nikon, Japan) as previously described [57, 58].

### Metabolite analyses

Supernatant was collected from culture vessel and culture plates; glucose, glutamine, lactate, and ammonia concentrations were analysed using Bioprofile 100 plus (NOVA Biomedical, USA). Specific metabolite consumption/production rates were calculated as described previously [33, 58].

### Cell cycle analyses

Cell cycle analyses were performed as previously described [32] using Nucleocounter NC-3000. Briefly, 1–3 × 10^6^ of dissociated hiPSCs were fixed with 70% ethanol in PBS for 2 h at 4 °C following by incubation with 1 μg/mL DAPI (Sigma-Aldrich, USA) in 0.1% Triton-X (Sigma-Aldrich, USA)/PBS for 5 min at 37 °C. After incubation, 10 μL of sample was directly loaded into the Nucleocounter NC-3000 for DAPI count, size and intensity staining measurement (*n* = 3). Cell cycle plots were analysed with FlexiCyte™ software (Chemometec, USA) to calculate cell cycle states/phases in percentage of sub-G1, G1-, S-, G2/M-phase.

### Flow cytometry

Cell/MC aggregates were dissociated into single cells with TrypLE™ Express (Thermo Fisher Scientific, USA), and the dissociated cell/MC suspension was pipetted through a 40-μm nylon mesh Easystrainer® (Greiner Bio-One, Austria) to remove the MCs. Subsequently, the dissociated single cells were fixed in 4% paraformaldehyde solution in PBS (Fisher Scientific, USA) for 15 min at room temperature. Thereafter, the cells were incubated with primary antibodies (Supplementary Table 1) in 1% bovine serum albumin (BSA) (Sigma-Aldrich, USA) with 0.2% Triton X-100 (Sigma-Aldrich, USA) in PBS for 30 min. Then, the samples were washed with blocking buffer 1% BSA in PBS, followed by incubation for 20 min at room temperature in 1:500 dilution of Alexa Fluor 647 conjugated goat anti-mouse secondary antibody (Thermo Fisher Scientific, USA), Alexa Fluor 488 conjugated goat anti-rabbit secondary antibody (Thermo Fisher Scientific, USA) or Alexa Fluor 647 donkey anti-rabbit secondary antibody (Thermo Fisher Scientific, USA). Cells were washed with blocking buffer again and analysed on a flow cytometer (GUAVA easy Cyte 8HT, Millipore, USA) using standard filter sets for secondary antibodies. A minimum of 10,000 events were captured per sample. Death cell and cell debris were excluded. Analyses and gating were performed with Flowjo® 10.0.7 software (Tree Star Inc., USA).

### Immunocytochemistry analyses

Cell/MC aggregates were harvested on day 22 of the bioreactor process. They were washed and fixed with 4% paraformaldehyde for 10 min at room temperature and then blocked for 1 h in 3% BSA in PBS. Primary antibodies (Supplementary Table 1) were incubated overnight at 4 °C in 0.2% Triton X-100 and 3% BSA in PBS. Thereafter, Alexa Fluor 488, 594 or 647 conjugated goat anti-mouse/rabbit secondary antibodies were incubated for 2 h at 1:500 dilutions in 3% BSA/PBS. Cell imaging was performed with a Nikon eclipse *Ti*-E inverted fluorescence microscope (Nikon, Japan). Pseudo colours were added for a better visualization of the cyto-antibody stains with ImageJ® (NIH, USA).

### Electrophysiological characterization and data analysis

Cardiomyocytes were plated onto 27-mm glass bottom dish (Thermo Fisher Scientific, USA) pre-coated with 2% growth factor reduced Matrigel® (BD Bioscience, USA) and cell culture maintained in cardiomyocyte maintenance medium (RPMI-1640 with Glutamax, 1% 100× penicillin/streptomycin (Thermo Fisher Scientific, USA), 1% 100× sodium pyruvate (Thermo Fisher Scientific, USA), 200 μM l-ascorbic acid 2 phosphate sesquimagnesium salt hydrate (Sigma-Aldrich, USA), 2% B27 supplement (Thermo Fisher Scientific, USA). The media was changed every 3 days. Cultures were used for patch clamp recordings 4–8 days following cardiomyocyte attachment.

Spontaneous cardiac action potentials (sAPs) were obtained using perforated patch recordings by means of a patch-clamp amplifier (EPC-9, HEKA Elektronik, Germany) with 20 KHz sampling rate controlled by Pulse software (HEKA Elektronik, Germany). All experiments were performed at physiological temperature (35–37 °C) constantly maintained by a TC-344B dual channel heating system (Warner Instruments, USA). Patch pipettes were pulled from borosilicate glass (Science Products GmbH, Germany) on a P-97 micropipette puller (Sutter Instrument, USA). Open tip pipette resistance was 3–6 MΩ. During measurement, cells were continuously perfused with a 37 °C bath solution containing 140 mM NaCl, 5 mM KCl, 1.8 mM CaCl_2_, 1.0 mM MgCl_2_, 10 mM glucose and 10 mM HEPES (pH 7.2; NaOH). The gramicidin-perforated patch pipette solution contained 70 mM K-Aspartate, 75 mM KCl, 7 mM NaCl, 10 mM HEPES, 1 mM MgCl_2_, 5 mM Mg-ATP, 0.3 mM Na-GTP and 30 μg/mL Gramicidin (pH 7.2; KOH). All reagents were purchased from Sigma-Aldrich, USA, unless otherwise specified. Concurrently, fluorescence-based optical phenotyping of CMs was performed by using a voltage sensitive FluoVolt dye (Thermo Fisher Scientific, USA), and the absolute fluorescence signals were measured using a custom built novel high-throughput OptioQUANT*™* platform (Ternion Bioscience, Singapore).

All off-line analysis was performed with Igor Pro (WaveMetrics, USA). Cell membrane capacitance, sodium current amplitude at − 20 mV, AP amplitude, peak voltage, resting membrane potential, maximal rate of depolarization and AP duration at different levels of repolarization (APD measured at 10%, 30% and 90% decrement of AP amplitude; at 0 mV during repolarization phase) were obtained. Data from cells in which the APD90 has more than 10% run-down were discarded. Cardiomyocytes were phenotyped using APD_80–70_/APD_30–20_ ratio. All values are given as mean ± SD.

### Statistical analyses

For comparison between two data sets, significance was calculated by Bonferroni corrected Student’s *t*-test. For comparison between multiple data sets, significance was calculated by Bonferroni corrected one-way ANOVA test. Error bars indicated on figures represent ± standard deviations (SD) of at least three repetitions.

## Results

### Selection of high cardiac differentiation potency hiPSC line in monolayer cultures

Five (DF6, CB6, BM-1, IMR90 and FR202) hiPSC lines were evaluated for cardiac differentiation efficiency in six-well plates using protocol based on the induction with 2 Wnt modulators, CHIR99021 and IWR-1, as previously reported [32, 40, 41, 58]. On day 14, the expression of cardiac markers NKX2-5, Troponin T and MLC2a; mesenchymal marker CD44; and endodermal marker hepatocyte nuclear factor 4 alpha (HNF4a) were measured. Results show that the expression of cardiac differentiation markers (NKX2-5, Troponin T and MLC2a) were low in DF6 (22–35%) and CB6 (1.7–2.1%) (Table 1A). They also showed ~ 40% CD44 expression in these lines indicating the potential for fibroblastic, mesenchymal differentiation (Table 1A). Moreover, DF6 showed ~ 40% HNF4a expression indicating that those cells were differentiated towards definitive endoderm, rather than towards cardiac mesoderm. Thus, DF6 and CB6 lines were not considered for further for bioprocess development. BM-1, IMR90 and FR202 showed a consistent high cardiogenic expression of NKX2-5 (60–80%), Troponin T (80–83%) and MLC2a (65–70%), and thus, they were selected for further for testing in stirred MC spinner culture.

**Table 1:** Selection of hiPSC lines regarding their expansion compatibility and cardiac differentiation efficiency in MC cultures. (A) Measurement of the highest flow cytometry population percentage of NKX2-5, Troponin T, MLC2a, CD44 and HNF4a on day 14 with a 24 h CHIR99021 induction of 4–14 μM on 5 cell lines in a monolayer attachment culture (*n* = 3). (B) Effects of continuous stirring on cell lines in MC-based spinner flask cultures. Cell yield, aggregate size and the expression of pluripotent (Oct4a and Tra-1-60) were measured (*n* = 3). (C) Selection of the best cell line for the cardiac differentiation on MC in a stirred spinner. Cell yield and the expression of cardiac marker Troponin T were measured (*n* = 3)

	DF6	CB6	BM1	IMR90	FR202
(A) **Cardiac differentiation efficiency with CHIR99021 in MNL cultures (Maximum flow cytometry population expression at 4-14 μM CHIR99021 on day14)**					
NKX2–5 (%)	21.8 ± 17.1	1.7 ± 0.1	78.8 ± 25.5	82.9 ± 8.4	57.1 ± 7.2
Troponin T (%)	29.7 ± 24.6	2.1 ± 0.4	81.0 ± 31.2	83.1 ± 8.9	80.6 ± 2.1
MLC2a (%)	34.9 ± 25.7	1.95 ± 0.3	70.4 ± 21.9	64.9 ± 0.1	64.9 ± 9.4
CD44 (%)	40.5 ± 9.2	32.1 ± 8.4	16.5 ± 11.7	37.1 ± 14.9	3.7 ± 3.7
HNF4a (%)	38.8 ± 14.8	7.4 ± 1.9	13.6 ± 1.5	20.7 ± 5.9	4.4 ± 4.4
(B) **Cell growth on MC in stirred spinner cultures (day 6)**					
Cells/mL (×10^6^)			No cell growth	1.7 ± 0.3	1.9 ± 0.6
Expansion fold				14.0 ± 0.2	16.0 ± 0.5
Aggregate size (mm^2^)				0.42 ± 0.1	0.30 ± 0.1
Oct4a				94.3 ± 1.1	91.0 ± 0.1
Tra-1-60				93.0 ± 0.01	96.4 ± 0.1
(C) **Cardiac differentiation on MC in stirred spinner cultures (day 12 of differentiation)**					
Cells/mL (×10^6^)				2.1 ± 0.4	2.3 ± 0.2
Troponin T (%)				14.4 ± 8.5	83.2 ± 0.13
CM yields (cells/mL × 10^6^)				0.32 ± 0.2	1.9 ± 0.03

### Testing of cell expansion and cardiomyocyte differentiation in stirred MC cultures

**Fig. 2:**
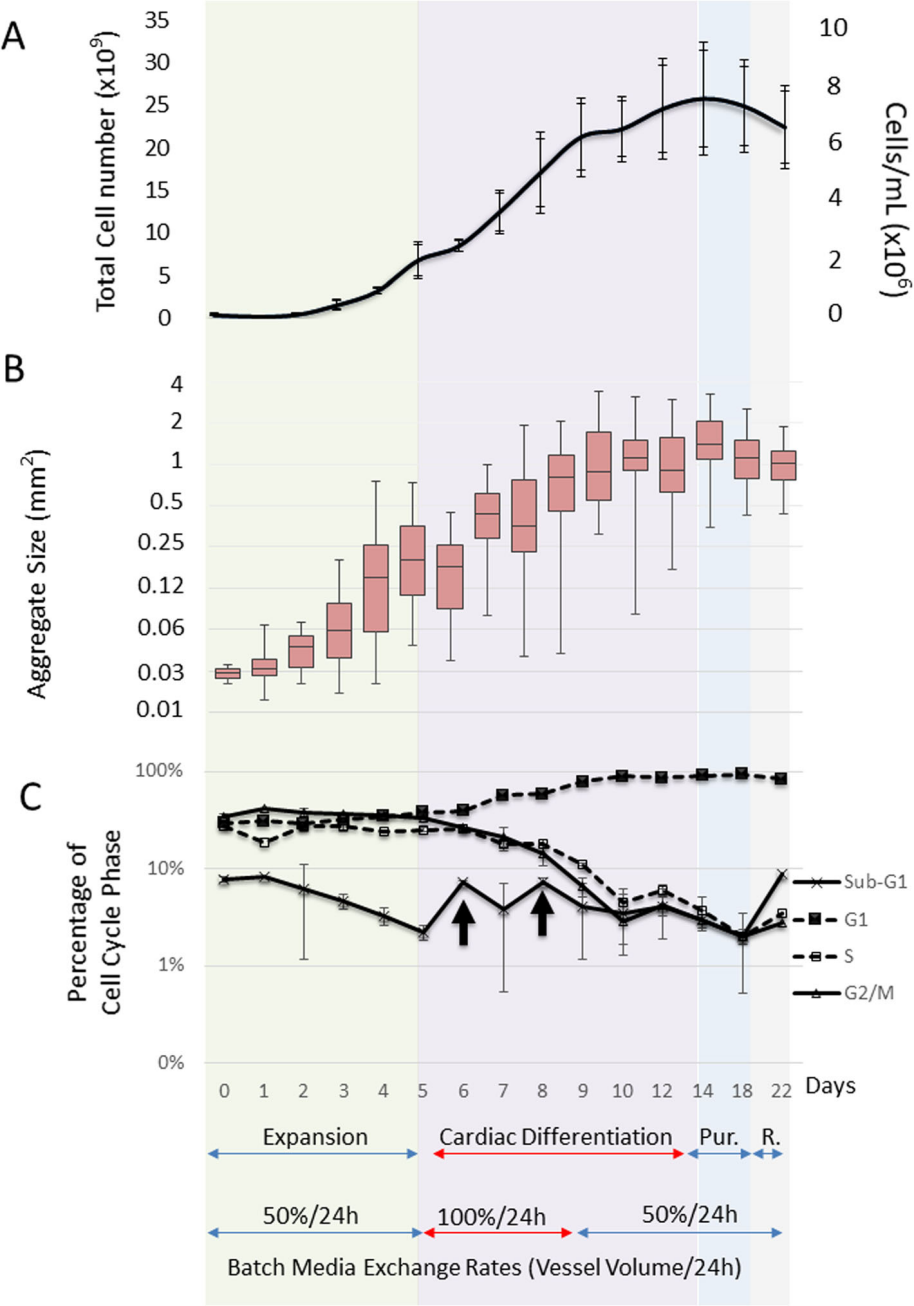
Cell growth, aggregate size and cell cycle changes during the integrated bioprocess. **a** Measurements of the total cell count of cell/MC aggregates (*n* = 3) by Nucleocounter. Graph represents as total and cell/mL. **b** Boxplot of the visual imaging measurements of the MC aggregate size (*n* = 150). **c** Illustration of the cell cycle percentage of the Sub-G, G1, S and G2/M phases (*n* = 3). Arrows indicate increase in cell in sub-G1 phase (*n* = 3 independent bioreactor runs)

BM-1, IMR90 and FR202 cell lines were selected for further adaptation to a MC spinner culture under continuous stirring (25 rpm) over 6 days. Images in Supplementary Figure 1 showed that BM-1 cells did not attached on the Geltrex®-coated Cytodex 1, and eventually formed suspension aggregates in the continuous stirring culture. On the other hand, IMR90 and FR202 were attached and significantly expanded (14-fold and 16-fold, respectively, Table 1B) in the MC culture under continuous stirring. Both cell lines formed cell/MC aggregates with sizes 0.3–0.4 mm^2^ in the 6 days’ culture (Table 1B). For instance, Supplementary Figure 3 evidently showed all MCs were covered with FR202 cells after 2-h post-seeding (Attachment D0). Then, cell/MC aggregates were formed and expanded throughout the process from day 1 to day 5. The aggregate size was measured and images were taken during the expansion phase (Fig. 2b). As in our previous report [36], the aggregates changed in size over time was due to cell proliferation, and subsequently free MC were being engulfed into the pre-existing proliferating hESC on the MC through a phenomenon that is putatively regulated by hESC migration. Finally, spreading is visible and aggregates up to 2 mm sizes are formed. And so, IMR90 and FR202 were chosen for further cardiac differentiation in stirred MC spinner cultures.

After 6 days’ expansion phase, IMR90 and FR202 cells on MCs were directly subjected to cardiac differentiation by CHIR99021 induction under continuous stirring (25 rpm) in a spinner flask (50 mL). Similar to our previous reports [57, 58], IMR90 line showed very low CM yields (0.32 ± 0.2 × 10^6^ cells/mL) in a stirred spinner flask under continuous stirring. This low cell yield is attributed to shear sensitivity of cells during differentiation which requires application of intermittent agitation which is problematic in scaling up of processes. Remarkably, FR202 showed high CM yields even when continuous agitation was applied (1.9 ± 0.03 × 10^6^ cells/mL and purity of 83.2 ± 0.13 cTnT%; Table 1C).

Collectively, out of 5 tested cell lines, the FR202 was selected for the cardiac bioprocess due to its high cardiac potential (Table 1A), expansion potential on MCs (Table 1B) and high CM production in continuously stirred MC spinner cultures (Table 1C).

### Integrated cardiac differentiation bioprocess in a stirred tank bioreactor

The integrated cardiac differentiation bioprocess consists of four phases (Fig. 1): expansion phase (5 days), differentiation phase (9 days), purification phase (5 days) and recovery phase (3 days). Figure 2 shows the progression of (A) cell growth, (B) cell/MC aggregate sizes and (C) cell cycle phases during the integrated bioprocess. The averaged outcomes of the three independent bioreactor runs are depicted.

During the expansion phase, a 2-day lag-phase followed by cell exponential growth was observed (Fig. 2a). After the 5 days’ culture, ~ 16-fold cell expansion (maximum cell density reached about 2 × 10^6^ cells/mL in 300 mL mTeSR™1 expansion medium) with pluripotent marker expression of > 85% and cell/MC aggregates with sizes about 0.24 mm^2^ were generated (Table 2A). Glucose was consumed successive from 2.58 to 0.63 g/L (consumption rate ~ 0.39 g/L/day), and the lactate production was increased simultaneously from 0.8 to 1.8 g/L (production rate ~ 0.2 g/L/day) (Supplementary Figure 2A). A similar metabolic profile was observed with the glutamine consumption (~ 0.33 mmol/L/day) and ammonium production (~ 0.13 mmol/L/day) (Supplementary Figure 2B). The yield lactate/glucose ratio and glutamine/ammonium during the exponential growth phase is about 1.93 and 0.78, respectively (Table 2A). This indicates that cell respiration was mostly glycolytic [11].

**Table 2:** Integrated one-unit (A) expansion, (B) cardiac differentiation, (C) purification and (D) recovery process of hiPSC FR202 in a controlled stirred tank bioreactor. Three independent runs confirmed the robustness of this integrated process

(A) **Expansion phase**										
Duration	Cell expansion			Pluripotent markers (%)					Metabolic yield ratio	
	Viable cells (cells/mL)	Cell expansion fold	Cell size (mm^2^)	OCT4a	Tra-1-60		Nanog		Y_Lac/Gluc_	Y_Amm/Gln_
5 days	1.94 ± 0.6 × 10^6^	16.1 ± 5.2	0.24 ± 0.1	87.6 ± 0.04	95.1 ± 0.04		84.7 ± 0.1		1.93 ± 0.1	0.78 ± 0.02
(B) **Differentiation phase**										
Duration	Cell expansion			Cardiomyocyte markers (%)				Cardiomyocytes yield (CM/mL)	Metabolic yield ratio	
	Viable cell count (cells/mL)	Cell expansion Fold	Cell size (mm^2^)	Troponin T	MF20	MLC2a	NKX2–5		Y_Lac/Gluc_	Y_Amm/Gln_
9 days	7.01 ± 0.2 × 10^6^	3.6 ± 0.9	1.19 ± 0.8	67.9 ± 0.1	72.4 ± 0.3	63.4 ± 0.1	60.9 ± 0.1	4.6 ± 0.03 × 10^6^	1.43 ± 0.1	0.68 ± 0.01
(C) **Purification phase**										
Duration	Cell expansion			Cardiomyocyte markers (%)				Cardiomyocytes yield (CM/mL)		
	Viable cell count (cells/mL)		Cell size (mm^2^)	Troponin T	MF20	MLC2a	NKX2–5			
5 days	7.1 ± 1.6 × 10^6^		1.15 ± 0.5	83.2 ± 0.1	79.6 ± 0.2	58.9 ± 0.1	63.1 ± 0.1	5.9 ± 0.01 × 10^6^		
(D) **Recovery phase**										
Duration	Cell expansion			Cardiomyocyte markers (%)				Cardiomyocytes yield (CM/mL)	Yield (CM/hiPSCs)	
	Viable cell count (cells/mL)		Cell size (mm^2^)	Troponin T	MF20	MLC2a	NKX2–5			
3 days	6.4 ± 1.4 × 10^6^		1.06 ± 0.4	96.4 ± 0.01	95.5 ± 0.1	55.8 ± 0.1	41.7 ± 0.1	6.2 ± 0.02 × 10^6^	41.1 ± 3.7	

Cardiac differentiation was then induced by simply changing the expansion medium into differentiation medium (RPMI/B27^-IN^) with 12 μM CHIR99021 in the bioreactor. The stirring speed was initiated at 33 rpm and increased to 40 rpm over time to avoid aggregates settlement and to control the size of the aggregates. After the IWR-1 induction on day 5 of the differentiation phase, the air mixture was changed to O_2_/Air to maintain a minimum of 30% DO saturation (hypoxia condition) [58], and there was an overall 3.6-fold expansion (cell density of about 7 × 10^6^ cells/mL) with more than 80% of Troponin T expression over the 9-day cultures (Table 2B and C). Beating aggregates were observed on day 11 of the bioprocess (Supplementary Video 1). Supplementary Video 2 shows the beating aggregates on day 14 after cardiac differentiation. CHIR99021 and IWR-1 inductions led to cell losses indicated by the increase in sub-G1 cell types of the cell cycle (Fig. 2c, arrows), illustrating the moderate cytotoxicity of these two Wnt modulators. The transition from exponential growth into the plateau phase (Fig. 2a) coincides with a change in cell cycle, demonstrated by a steep decline of the proliferative S, G2/M phases and an increase of the G1 phase between days 9–10 (Fig. 2c). The aggregates sizes were also increased from 0.24 to 1.2 mm^2^ over the differentiation phase (Table 2A and B and Fig. 2b). The molar ratio of produced lactate to consumed glucose, Y_Lac/Gluc_, is low (1.4 ± 0.1, Table 2B) suggesting that the differentiated cells tend to Krebs cycle via aerobic metabolism as compared with cell expansion phase which is mainly via anaerobic metabolism, consistent with previous reports [21, 33]. High glucose consumption (0.68 g/L/day) and lactate production (0.5 g/L/day) were observed from day 5 to day 8 indicating a high metabolic rate and proliferation during the early differentiation phase (Supplementary Figure 2A). For that reason, a 100% media change per day during day 5 to day 8 was carried out. After day 8, 50% media change per day was then performed. A cell density of 7.01 ± 0.2 × 10^6^ cells/mL with about 70% Troponin T was achieved (Table 2B).


**Additional file 5:** Supplementary Video 1. Video image of the FR202 cell line on Cytodex 1 from a stirring tank bioprocess culture on day 11 during the bioprocess demonstrating the onset of contractile beating.



**Additional file 6:** Supplementary Video 2. Video image of the FR202 cell line on Cytodex 1 from a stirring tank bioprocess culture on day 14, end of the differentiation phase.


On day 14 of the bioprocess (after 5 days expansion and 9 days differentiation), cardiomyocyte purification was carried simply by changing the differentiation medium into a metabolic restricting medium (purification medium; RPMI + 4 mM lactate, without glucose and pyruvate) [58] for purification (Fig. 1). Subsequently, the culture medium was changed back to differentiation medium (RPMI/B27^-IN^, with glucose and pyruvate) for cell recovery (Fig. 1). Supplementary Video 3 and 4 show the beating aggregates on day 18 end of purification phase and day 21 end of recovery phase, respectively. The cell density was reduced to 6.4 ± 1.4 × 10^6^ after recovery phase (Table 2D) due to the loss of the non-cardiomyocytes; however, the purity was significantly improved, from ~ 68 to ~ 96% of Troponin T (Table 2B–D). Despite the lower cell density and reduced aggregate size (1.19 to 1.06mm^2^, Table 2B–D), there was an increase in the overall cardiomyocyte yield from 4.6 × 10^6^ to 6.2 × 10^6^ CM/mL (Table 2C and D), since ~ 96% of Troponin T positive cells were obtained after the recovery phase.


**Additional file 7:** Supplementary Video 3. Video image of the FR202 cell line on Cytodex 1 from a stirring tank bioprocess culture on day 18 end of the purification phase.



**Additional file 8:** Supplementary Video 4. Video image of the FR202 cell line on Cytodex 1 from a stirring tank bioprocess culture on day 22 end of the recovery phase.


### Gene expression kinetics of during FR202 cardiac differentiation in stirred microcarrier bioreactor

**Fig. 3:**
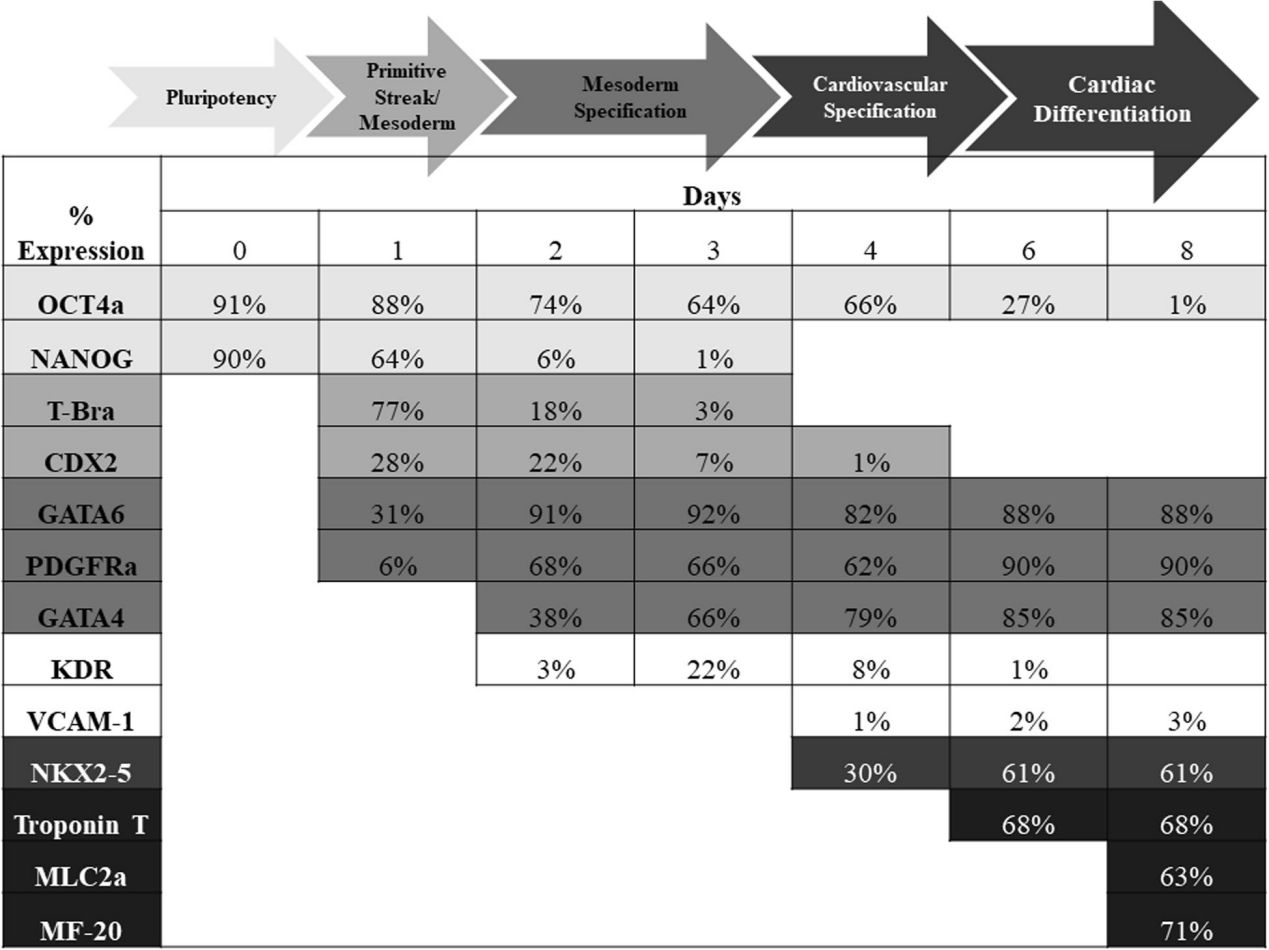
Flow cytometry population percentage analyses of pluripotency markers (OCT4a, NANOG), mesoderm markers (T-Bra, CDX2, GATA6, GATA4 and PDGFRa), cardiovascular markers (KDR, VCAM-1 and NKX2-5) and early cardiac markers (Troponin T, MLC2a) during cardiac differentiation over 8 days (*n* = 3 independent bioreactor runs)

**Fig. 4:**
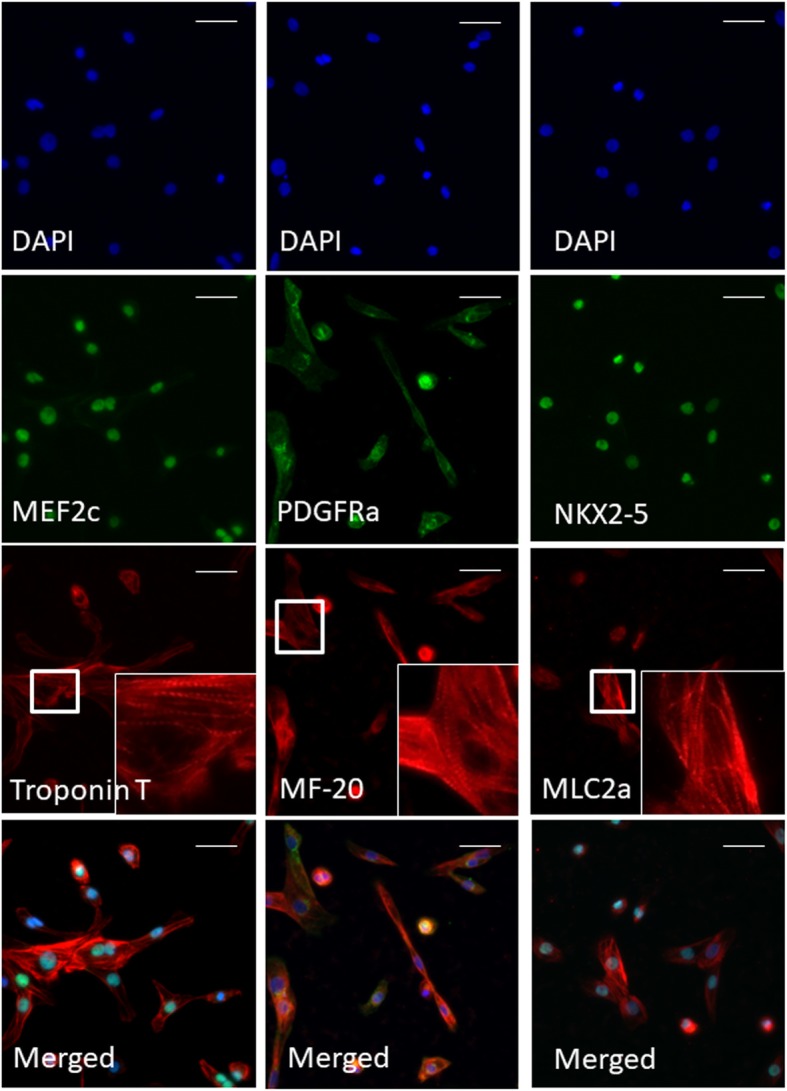
Immunocytochemistry of FR202 cells after expansion, differentiation, purification and recovery phases in stirred tank bioreactors. Cells were harvested on day 22 and re-plated on Geltrex®-coated plates. Images show nuclear localization of MEF2c and NKX2-5, cytosolic localization of PDGFRa and structural localization of Troponin T, MF-20 and MLC2a. Magnification of Troponin T, MF-20 and MLC2a area shows the sarcomere structures. Scale bar = 20 μm

On day 1 of cardiac differentiation, a transient increase in T-bra and CDX2 was observed indicating primitive streak development, whereas pluripotent NANOG-positive population dropped by ~ 30% (Fig. 3). Mesoderm specification was observed during day 1 to 3 with an increase and saturation in successive order of GATA6, PDGFRa and GATA4. Pluripotency marker NANOG was lost and OCT4a was decreased to 66 ± 3%. Cardiovascular specification was initiated on day 4 by the onset of NKX2-5 expression. Cardiac differentiation phenotype was established during days 6–8 with expression of Troponin T (~ 68%), MLC2a (~ 63%) and MF-20 (71%) and led to a reduction of pluripotency marker OCT4a to ~ 1%. The differentiated FR202 cells were negative for other non-cardiovascular lineages, such as blood lineage KDR expression or endothelial cell marker VCAM-1 expression which were transient and limited. The structural expression of Troponin T, MLC2a and MF-20 showed the characteristic segments of the actin filaments (Fig. 4). Also, nuclear expression of NKX2–5 co-localized with MLC2a expression. Nuclear MEF2c expression co-localized with Troponin expressions. Cytosolic PDGFRa expression co-localized with MF20 expression.

### Electrophysiological characterization and phenotyping of FR202-derived CMs

**Fig. 5:**
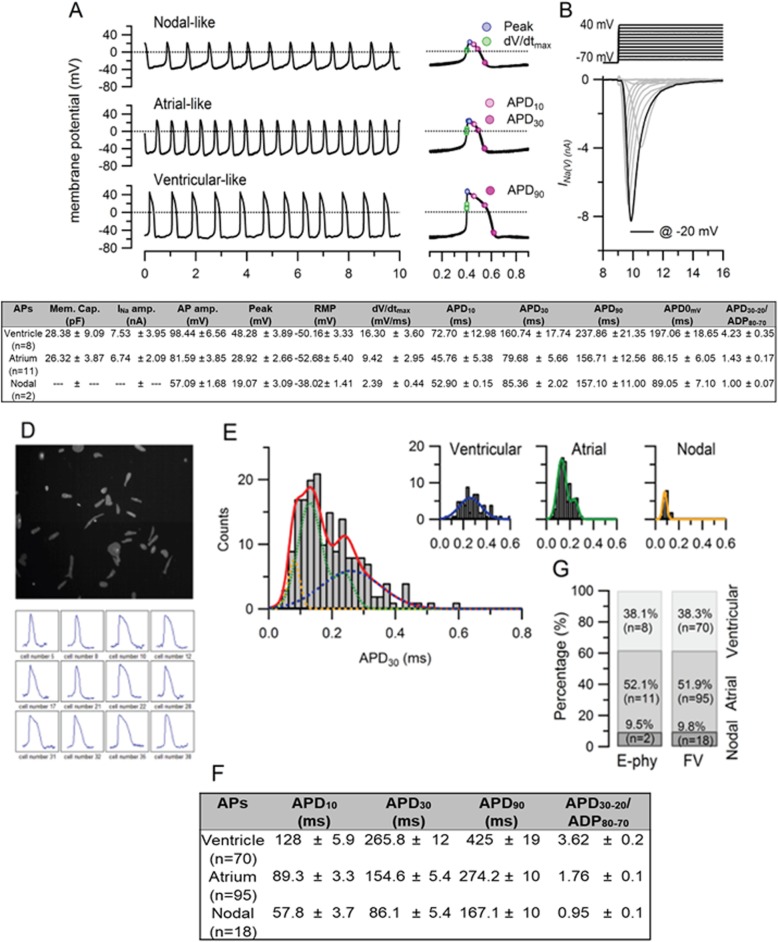
Cardiac electrophysiology. Spontaneous action potentials (sAPs) were recorded from 21 cardiomyocytes at 35–37 °C using the perforated patch-clamp method. **a** Recording of resting membrane potential, representative recordings showed 3 major action potentials types: nodal-like (upper), atrial-like (middle) and ventricular-like (bottom). Individual action potentials were aligned at the half-rise of the upstroke phase and shown at an expanded time scale (right). Dotted lines indicate 0 mV. **b** Representative INa(V) traces as the result of the applied voltage protocol. The black trace indicates the current recorded at − 20 mV. **c** Summary table displays cell membrane capacitance, the peak amplitude of INa(V) at − 20 mV and sAP parameters. **d** Low-density seeded hSC-CMs monolayer loaded with the FlouVolt (FV) dye and observed at 520 nm (upper). The relative fluorescent light value was calculated over time for each region of interest (ROI). Twelve representative AP traces are shown here (bottom). **e** Histograms of APD30 calculated from each ROI with bin size 20 ms. The red line indicates the total of three binomial Gaussian distributions (dashed line) which is the sum of the three histograms of atrial, ventricular and nodal phenotypes shown in insert figures. **f** Summary table displays sAP parameters obtained from 183 waveforms. **g** The bar graph shows the percentage of each cell type based on the parameters of sAP recorded by electrophysiological and optical approaches. AP amp., AP amplitude; Peak, peak voltage; RMP, resting membrane potential; dV/dtmax, maximal rate of depolarization; APD, AP duration at different levels of repolarization (APD measured at 10%, 30% and 90% decrement of AP amplitude); APD30-20/ APD80-70, APD ratio during phase 2.

Spontaneous action potential (sAP) of FR202-derived CMs were recorded by two different methods: patch electrodes recording signal using digitizer and optical imaging using voltage-sensitive dye FluoVolt (Fig. 5). Example sAP of 21 tested CMs recorded by conventional patch electrodes are shown in Fig. 5a. Three major types of cardiac AP phenotypes (nodal-like, atrial-like, and ventricular-like) were recorded. Activation of inward sodium current (INa) was observed at voltage + 40 mV to − 70 mV, and the maximum amplitude was obtained at pulse to − 20 mV (Fig. 5b). To further identify different cardiac phenotypes of the FR202-derived CMs, the AP-related parameters, such as maximal rate of depolarization (dV/dtmax) and AP duration (APD) at different levels of repolarization (10%, 30% and 90%) are were also quantified (Fig. 5c). Smaller AP amp (57.09 ± 1.68 mV) with lower dV/dtmax values (2.39 ± 0.44 mV/ms) were categorized as nodal-like (pace maker). A minimal plateau phase (APD_30–20_/ADP_80–70_ < 1.5) with shorter duration APD_0mv_ (86.15 ± 6.05 ms) were categorized as atrial-like CMs. Ventricular-like CMs have longer duration APD_0mv_ (197.06 ± 18.65 ms) with a plateau phase (APD_30–20_/ADP_80–70_) about 4.23 ± 0.35. Those are the criteria used to distinguish the phenotypes of hiPSC-derived CMs [19, 42]. Most of the differentiated CMs were atrial-type cells, with 52.1% of the 21 CMs tested displayed atrial-like APs, 38.1% showed ventricular-like APs, and 9.5% showed nodal-like APs (Fig. 5g). Figures 5 d to f illustrate that the FluoVolt dye was also able to measure the AP phenotypes, with overall higher throughput (*n* = 183) at a shorter time compared to patch clamp. The phenotypes can be clearly identified as ventricular-like (APD_30–20_/ADP_80–70_ = 3.6 ± 0.22) and atrial-like (APD_30–20_/ADP_80–70_ = 1.76 ± 0.07), and comparable to the conventional patch clamp approach. The same percentage of nodal-like, atrial-like, and ventricular-like phenotypes were identified using the FluoVolt dye with the OptioQUANT™ platform (Fig. 5g).

In summary, hiPSC FR202 was selected for cardiac differentiation in an integrated MC-based bioreactor system, as its showed higher compatibility for expansion and differentiation on microcarriers under continuous stirring conditions. After a 22-day bioprocess in a controlled bioreactor, high-quantity (~ 1.9 × 10^9^ CM; ~ 40 CM/hiPSC seeded, Table 2D) and high-quality (> 96% Troponin T, Table 2D), atrial-, ventricular- and nodal-like (Fig. 5) cardiomyocytes were produced.

## Discussion

The goal of our study is to develop a scalable bioprocess for production and purification of cardiomyocytes from hiPSCs in a stirred tank bioreactor. The combination of large quantities of cells from a bioreactor and purification of homogeneous cardiac population facilitates the transplantation of CMs derived from hiPSC for heart disease in the future. Selection of a hiPSC line with high potential for expansion and cardiac differentiation on MCs is essential. It was apparent that not all hiPSC lines can expand in MC culture and then differentiate into the cardiac lineage (Table 1). Cell line-to-line variations occurred [25, 32], possibly during the reprogramming phase of the iPSCs which are derived from various sources. IMR90 was reported earlier as an easy cell line with high differentiation potential in 2D monolayer or suspension EBs [23]; however, IMR90 is also sensitive to agitation. Applying intermittent agitation during the first 3 days of cardiac differentiation phase is necessary [57, 58], yet this is problematic to implement in large-scale reactors. FR202 was then selected as it showed a higher compatibility for expansion and cardiac differentiation in our MC-based stirred suspension bioreactor. Overall, our process yielded > 40 CMs per seeded hiPSC (Table 2D), which is at least eightfold more than other reports. They ranged from 1 to 5 CMs/hPSC [10, 13, 14, 27, 28, 38, 57, 60], and 35% more than our previous report [58]. This difference in CM yields is mainly attributed to the high proliferative capacity in the initial expansion phase (~ 16-fold) and differentiation phase (~ 3-fold), as well as > 96% of Troponin T obtained after the recovery phase. It is likely possible to further increase the purity of CMs if the culture is extended for 1–2 days after purification; however, the CM yields may reduce as predicted from Fig. 2a. To the best of our knowledge, this is the first report demonstrating the generation of high-quantity (> 40 CM/hiPSC) and high-quality (> 96% Troponin T) cardiomyocytes on MC-based platform in one continuous process under a controlled bioreactor.

The limitation to shear force could be a challenge for the scale up of the cardiac differentiation processes [39, 57, 58]. Moreover, during the small molecule supplementation of the differentiation process, aggregates easily break apart, which can lead to a high loss of cells [33, 57, 58]. Thus, the stirring speed in the bioreactor was adjusted empirically (33 to 40 rpm) to keep the aggregates suspended at a minimal shear rate and to control the aggregates to certain sizes 200–800 μm in diameter, i.e. 0.13 to 2 mm^2^ [33, 47, 58]. Other shear protective strategies such as increase in media viscosity and single cell protective reagents (e.g. Y-27632 Rock inhibitor) could be important in scale-up development. The MC aggregates generated during the bioprocess grew from ~ 0.24 mm^2^ in the expansion phase to ~ 1 mm^2^ after differentiation (Fig. 2b and Table 2). These values are in agreement with the size of aggregates obtained in previous reports [33, 47, 58].

One of the main bioprocess advantages of the MC technology is the ability to rapidly sediment the aggregates allowing 100% media changes via an elevated dip tube without significant loss of aggregates [7, 8]. We used this method to wash and dilute media and adjust small molecule concentrations during the differentiation phases. The residual concentration of mTeSR1™, CHIR99021 and IWR-1 are of concern during the differentiation phase, because these compounds can alter differentiation pathways. We reported previously that residual concentration of up to 1.5 μM CHIR99021 can be tolerated during the differentiation process [32]. Changing medium during a bioprocess can be cumbersome and requires often offline cell washes and product manipulation. Our media change strategy avoided additional processing steps by keeping a low residual media volume in the stirred tank bioreactor during all phases. In order to improve cell viability, as well as minimize medium utilization, optimization of the feed rate was accomplished by employing metabolic activity-based feeding, based on the cells’ glucose consumption and lactate production (Supplementary Figure 2A). Only 50% media changes were conducted per day during the expansion phase, since glucose was not a limiting factor. However, during the first few days of cardiac differentiation phase, glucose consumption and lactate production were significantly increased and hence required 100% media changes per day, in order to replenish the glucose and remove the lactate. After day 9 of differentiation, the glucose consumption and lactate production were reduced, and therefore, only 50% medium was changed per day. This feeding strategy was successful for both hiPSC expansion and cardiac differentiation.

The oxygen levels were maintained at 30% DO saturation to support the hiPSC growth during the expansion phase and early differentiation phase as shown in a previous publication [47]. Reports showed that hypoxia activated the expression of hypoxia inducible factor (HIF-1), which may indirectly enhance cardiomyocyte differentiation by the activation of a number of growth factors (such as VEGF) that have a synergistic effect on mesodermal cardiogenesis [18, 43, 49, 51]. Further work is required, however, to optimize DO level during the different phases of the bioprocess system in order to improve the overall efficiency of CM generation.

Corroborating our earlier studies [32], temporal characterization of differentiating cells revealed the gradual decrease in expression of pluripotency markers OCT4a and NANOG, the peak of mesoderm marker T-bra on day 1, the gradual increase in endo-mesoderm development GATA4, GATA6 and PDGFRa on day 2, and increase in cardiac markers NKX2-5, Troponin T, and MLC2a on day 6 (Fig. 3). T-bra is an early mesoderm marker and has been reported to have an expression peak at day 3 during cardiac differentiation [4, 5, 17]. Here, an early expression peak of T-bra on day 1 (Fig. 3) was observed indicating the early endodermal and mesodermal cell population. This phenomenon is further supported by the reciprocal inhibition between NANOG and CDX2 at day 1. The rapid downregulation of NANOG and upregulation of T-bra and CDX2 (major determinant of extraembryonic mesoderm) has been shown at the outset of primitive streak induction [9]. It is important that the correct primitive streak mesoderm induction and patterning is initiated for CM differentiation [44]. In summary, our process induced early high expression of primitive streak and mesoderm markers, followed by endothelial-mesenchymal transition and cardiovascular progenitor development until the end of CM differentiation (Fig. 3).

Traditionally, patch-clamp method and micro-electrode array have been used in analysing the spontaneous action potential (AP) and also the electrophysiological properties of CMs [20, 42, 52]. However, these systems are time consuming, have low throughput, and are analysing indirect readouts [3]. Recently, to facilitate the recording efficiency of the electrical activity of CMs, optimal imaging with voltage-sensitive dyes was used [3, 55]. In our studies, a high throughput platform (OptioQUANT™) developed by Ternion Bioscience, Singapore, was used to image and quantify subtle changes of membrane potentials in hPSC-/hiPSC-derived CMs, based on voltage-sensitive dyes (FluoVolt) and high-speed microscopy (Fig. 5d). This method for the automated quantification of AP waveforms of more than 100 CMs simultaneously, which is not achievable via manual or automated patch clamping in the same duration. As compared with the traditional patch-clamp method that we performed, the OptioQUANT™ system recorded similar percentage of ventricular-like, atrial-like, and nodal-like activity (Fig. 5g), thereby removing any inherent selective bias during patch clamping. Cells exhibiting atrial-like AP characteristics were predominant (> 50%) in the CM population (Fig. 5g). Taken together, the use of voltage-sensitive dyes together with high-throughput high-content platforms such as OptioQUANT™ will be invaluable in providing high-speed and precise action potential-guided phenotyping information of hPSC-CMs in any scale-up efforts for cell therapy and also for precision drug screening.

Maintaining hiPSC-CM survival after cryopreservation and thaw has been a significant challenge. Although current protocols for cryopreserving hiPSC-CMs have already been established by using CryStor CS10 (BioLife Solutions Inc.) [61], our preliminary results on cryopreservation and post-thaw efficacy were very low. To adequately address this question, more studies that directly compare the physiological properties of freshly derived and cryopreserved hiPSC-CMs by using other freezing solutions, such as STEM-CELLBANKER from Nippon Zenyaku Kogyo, Japan [56], and methods, such as addition of activin A and bone morphogenetic protein-4 [63], should be further explored.

In conclusion, in this study, we documented the cell line selection process where we tested 5 cell lines for cardiac differentiation potency in monolayer cultures followed by MC expansion ability under continuous stirring. FR202 cell line exhibited the highest pluripotency, cardiac differentiation efficiency and growth, and was then selected to be expanded and differentiated in stirred bioreactors. The 22-day-long process integrated all process phases of pluripotent stem cell expansion on MCs as well cardiac differentiation, purification and recovery. MC-based differentiation allowed a batch feeding strategy by aggregate sedimentation under oxygen-restricted conditions with continuous stirring. Our process documents the critical time points for cell growth and cell loss during small molecule inductions and media changes. This process achieved a high density of functional cardiomyocytes (1.9 × 10^9^ cells) with > 95% Troponin T and > 50% are atrial-type. To the best of our knowledge, there are no reports describing such a high production yield of hiPSC-derived cardiomyocytes per hiPSC input (> 40 CMs, Table 2D). Ultimately, the future direction of this work leads towards studies on how to transfer our platform into GMP-compliant facilities for the production of cardiomyocytes for clinical uses.

## Supplementary information

Additional file 1: Supplementary Figure S1. Selection of hiPSC lines regarding their expansion compatibility in MC cultures under continuous stirring conditions. Bright field images of 3 cell lines expanded in stirring speed of 25 rpm of on Cytodex 1 spinner culture (scale bar = 1 mm).

Additional file 2: Supplementary Figure S2. Metabolic consumption and production during the integrated bioprocess. (A) Graphical illustration of glucose consumption versus lactate production and (B) glutamine consumption versus ammonium production of FR202 cells during the expansion and differentiation bioprocess.

Additional file 3: Supplementary Figure S3. Representative bright field images of FR202 cells during the 22-day integrated expansion, differentiation, purification and recovery phases on Cytodex 1 in a stirring tank bioprocess culture (scale bar = 1 mm).

Additional file 4: Supplementary Table S1. List of antibodies used in the study.

## Data Availability

Not applicable.

## Abbreviations

AP amp: AP amplitude
AP: Action potential
APD: Action potential duration
CDX2: Caudal-type homeobox 2
CHIR: CHIR99021
CM: Cardiomyocytes
DAPI: 4′,6-Diamidine-2′-phenylindole dihydrochloride
DO: Dissolved oxygen
dV/dtmax: Maximal rate of depolarization
EB: Embryoid bodies
FACS: Fluorescence-activated cell sorting
GMP: Good manufacturing practice
hiPSC: Human-induced pluripotent stem cell
HNF4a: Hepatocyte nuclear factor 4 alpha
hPSC: Human pluripotent stem cell
IWR-1: Inhibitor of Wnt
KDR: Kinase domain receptor
MC: Microcarrier
MF-20: Myosin heavy chain
MEF2c: Myocyte enhancer factor 2c
MLC2a: Myosin light chain 2 alpha
NKX2-5: NK2 homeobox 5
OCT4: Octamer-binding transcription factor 4
PBS: Phosphate-buffered saline
PDGFRa: Platelet-derived growth factor receptor alpha
sAPs: Spontaneous cardiac action potential
T-bra: T-Brachyury
VCAM-1: Vascular cell adhesion protein 1
