# Supplementary figures and images for: Selection of human induced pluripotent stem cells lines optimization of cardiomyocytes differentiation in an integrated suspension microcarrier bioreactor
Source: Stem Cell Res Ther. 2020 Mar 13;11:118. doi: 10.1186/s13287-020-01618-6 (PMC7076930; doi:10.1186/s13287-020-01618-6)

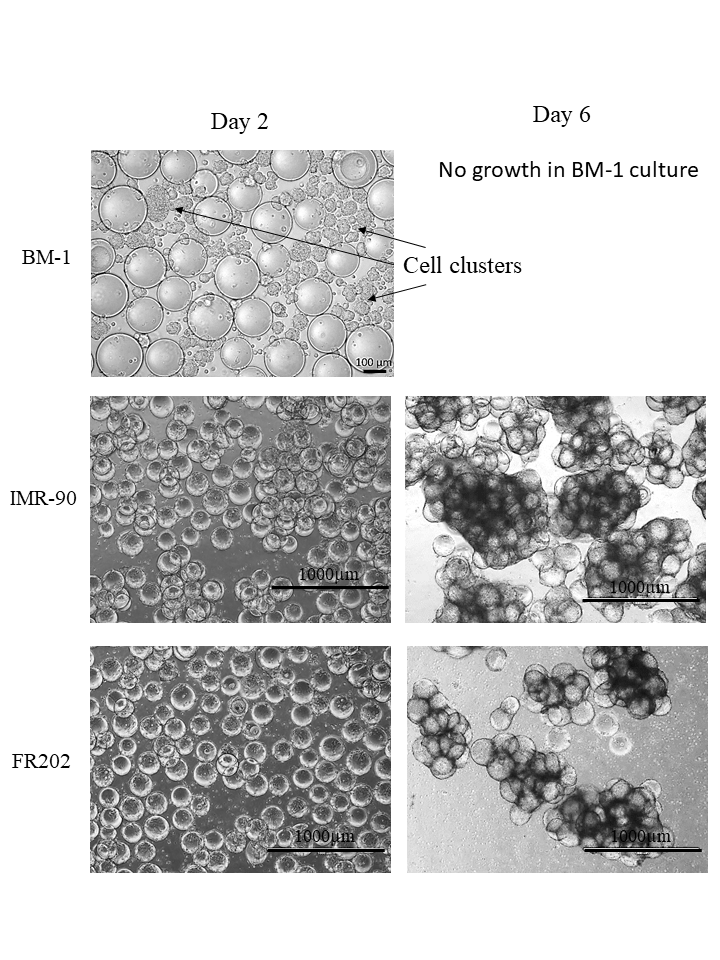

Supplement: Supplementary file 5 — Supplementary Figure S1. Selection of hiPSC lines regarding their expansion compatibility in MC cultures under continuous stirring conditions. Bright field images of 3 cell lines expanded in stirring speed of 25 rpm of on Cytodex 1 spinner culture (scale bar = 1 mm). [file 13287_2020_1618_MOESM1_ESM.tif]

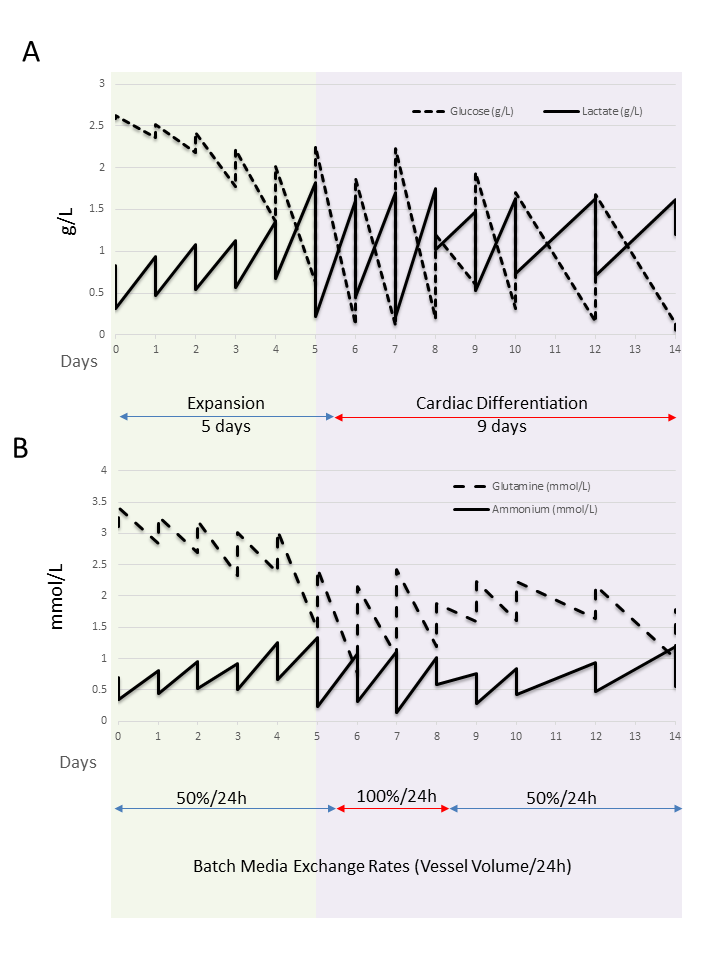

Supplement: Supplementary file 6 — Supplementary Figure S2. Metabolic consumption and production during the integrated bioprocess. (A) Graphical illustration of glucose consumption versus lactate production and (B) glutamine consumption versus ammonium production of FR202 cells during the expansion and differentiation bioprocess. [file 13287_2020_1618_MOESM2_ESM.tif]

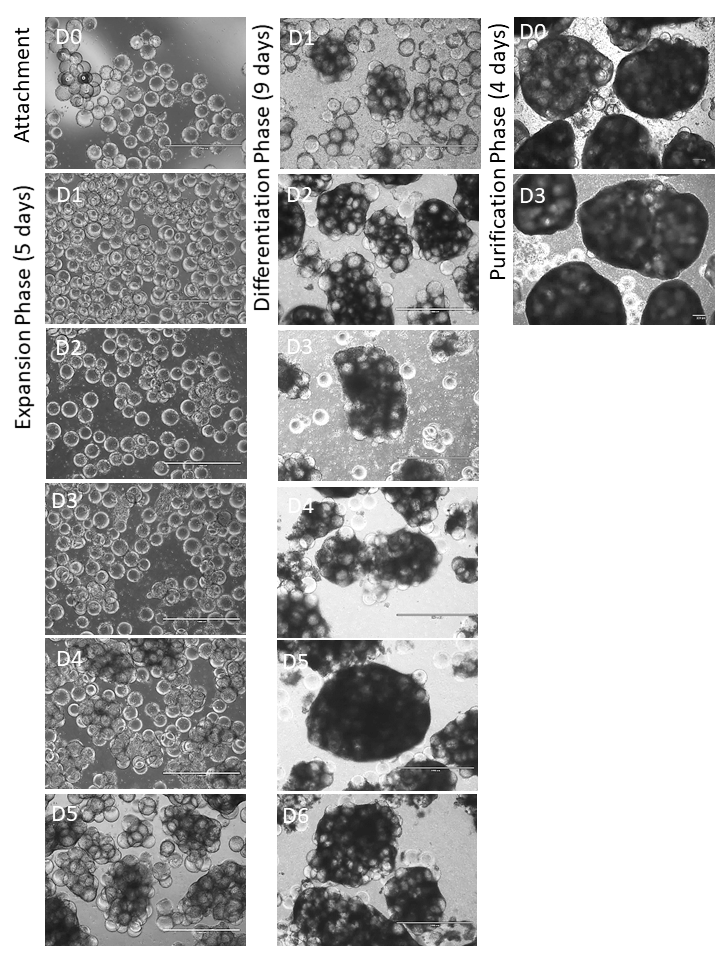

Supplement: Supplementary file 7 — Supplementary Figure S3. Representative bright field images of FR202 cells during the 22-day integrated expansion, differentiation, purification and recovery phases on Cytodex 1 in a stirring tank bioprocess culture (scale bar = 1 mm). [file 13287_2020_1618_MOESM3_ESM.tif]
