# Supplementary material for: Selection of human induced pluripotent stem cells lines optimization of cardiomyocytes differentiation in an integrated suspension microcarrier bioreactor
Source: Stem Cell Res Ther. 2020 Mar 13;11:118. doi: 10.1186/s13287-020-01618-6 (PMC7076930; doi:10.1186/s13287-020-01618-6)
Supplement: Supplementary file 8 — Supplementary Table S1. List of antibodies used in the study. [file 13287_2020_1618_MOESM4_ESM.docx]

Supplementary Table 1

| **Antibody** | Company | Catalogue number | Clone | Isotype | 2nd Ab | Expected kD | Dilution |
| --- | --- | --- | --- | --- | --- | --- | --- |
| **GATA4** | Cell Signalling | 36966 | D3A3M | IgG | rabbit | 55 | 1:200 |
| **GATA6** | Cell Signalling | 5851 | D61E4 | IgG | rabbit | 55 | 1:200 |
| **NANOG** | Cell Signalling | 4903 | D73G4 | IgG | rabbit | 42 | 1:300 |
| **NKX2.5** | Cell Signalling | 8792 | E1Y8H | IgG | rabbit | 30-42 | 1:150 |
| **PDGFRa** | Cell Signalling | 5241 | D13C6 | IgG | rabbit | 190 | 1:200 |
| **Oct4a** | R&D Systems | MAB17591 | 653108 | IgG2a | mouse |  | 1:400 |
| **SOX2** | Cell Signalling | 3579 | D6D9 | rabbit | rabbit | 35 | 1:400 |
| **T-Bra** | R&D Systems | IC2085G |  | IgG | mouse-conjugated |  | 1:50 |
| **TRA-1-60** | Cell Signalling | 4746 | TRA-1-60(S) | IgM | mouse | 200-400 | 1:100 |
| **Troponin T** | ThermoScientific | MA5-12960 | 13-11 | IgG1 | mouse |  | 1:400 |
| **MLC2a** | Synaptic Systems | 311011 | 56F5 | IgG | mouse |  | 1:800 |
| **CD44** | Cell Signalling | 3570 | 156-3C11 | IgG2a | mouse | 80 | 1:100 |
| **MF20** | DSHB |  |  | IgG2b | mouse | 223 | 1:100 |
| **CDX2** | Cell Signaling | 12306 | D11D10 | IgG | rabbit | 38 | 1:200 |
| **KDR** | Miltenyi Biotec | 130-093-598 | Avas12 | IgG2a | mouse-conjugated | 150 | 1:50 |
| **MEF2c** | Cell Signalling | 5030 | D80C1 | IgG | rabbit | 50-60 | 1:20 |
